# Clinicopathological features and immunohistochemical detection of antigens in acute experimental *Streptococcus agalactiae* infection in red tilapia (*Oreochromis* spp.)

**DOI:** 10.1186/2193-1801-2-286

**Published:** 2013-06-28

**Authors:** Syuhaidah Abdullah, Noraini Omar, Sabri Mohd Yusoff, Emikpe Benjamin Obukwho, Tanko Polycarp Nwunuji, Latifah Hanan, Jamil Samad

**Affiliations:** Department of Veterinary Pathology & Microbiology, Faculty of Veterinary Medicine, Universiti Putra Malaysia, 43400 UPM Serdang, Selangor Malaysia; Faculty of Veterinary Medicine, Universiti Putra Malaysia, 43400 Serdang, Selangor Malaysia

**Keywords:** Red tilapia, *Streptococcus agalactiae*, infection, IHC, PCR

## Abstract

This study investigates the clinicopathological features of acute experimental streptococcosis in red tilapia using various routes of infection; intraperitoneal (IP), immersion (IM) and immersion cut (IC). Twenty four red tilapia in duplicates were inoculated intraperitoneally with 10^9^ CFU/ml of *S. agalactiae* while another sets: intact, one with sharp cut at the tail end were exposed to bacterial inoculums 10^9^ CFU/ml diluted in water while two groups of control fish were similarly manipulated. Clinical signs were recorded; samples from the gills, brain, eyes and kidneys were also taken for bacterial isolation and histopathology. Immunohistochemistry (IHC) and polymerase chain reaction (PCR) were employed to detect the antigen. The diseased fish showed skin, fin haemorrhages and exophthalmia with obvious signs in IP at 2 hpc followed by IC and IM at 4 hpc. The lesions were noticed earlier in the kidney and most severe in IP. IHC detected antigen as early as PCR and isolation with intense staining in blood vessel lumen and wall, macrophages in choroid, focal haemorrhage in the renal interstitium and meninges especially in IP followed by IC and IM. The immunolocalisation of the antigen described for the first time further explain the pathogenesis of streptococcosis in red tilapia.

## Introduction

Tilapia *(Oreochromis* spp*.)* are reported to be among the most cultured food fish worldwide (Klesius et al. 2008). About 80% of the global farmed tilapia production in 2002 showed Asia as the largest world producer. Among the Asian countries, Malaysia is listed among the top ten producers of farmed tilapia (El-Sayed 2006). Tilapia, also known as “aquatic chicken” are fast growing with ability to survive in poor water conditions. They are white flesh, eat variety of food, easy to breed with simple and non-complicated hatchery technology (Nandlal and Pickering 2004). In terms of resistance, tilapia were also considered to be relatively resistant to bacterial, fungal, parasitic and viral diseases (Klesius et al. 2008).

Red tilapia (*Oreochromis niloticus* hybrid) were first introduced in mid-1980’s in Malaysia possibly due to their relative resistance to disease. In 1997, there was heavy mortality of about 300 to 400 g weight tilapia that were kept in floating net cages in Sungai Pahang (Siti-Zahrah et al. 2004), the fish showed clinical features of corneal opacity, exophthalmia, erratic swimming and occasional swollen abdomen, with mortality rate of 70%. Other similar outbreaks were recorded in cages of Kenyir, Pedu and Pergau Lakes in Malaysia in the mid year between April to July and the laboratory investigation conducted revealed the causative agent to be Gram-positive bacteria, *S. agalactiae* (Siti-Zahrah et al. 2005; Amal et al. 2008).

*S. agalactiae* had been identified as a pathogen responsible for causing infections in a wide range of hosts; chicken, camel, dolphin, horse, monkeys, cats, dogs, rabbits, nutrias, guinea pigs and mice (Garcia et al. 2008). It is well known to cause septicaemia and meningo-encephalitis in fish which include rainbow trout, seabream, tilapia, yellowtail, catfish spp., killifish, menhaden spp., mullet, and silver pompfret (Ferguson et al. 1994; Olivares-Fuster et al. 2008).

*Streptococcus* spp. has been reported to cause morbidity and mortality worldwide with estimated loss of about $150 million annually (Klesius et al. 2008). Among the variety of freshwater and saltwater fish species, *S. agalactiae* had been shown to cause high morbidity and mortality (Pasnik et al. 2006; Garcia et al. 2008). However, the pathogenesis of the infection in fish is still poorly understood (Eldar et al. 1995). In Malaysia, the outbreak of *S. agalactiae* has been reported as one of the emerging pathogen in tilapia culture (Abuseliana et al. 2011) and the clinicopathological changes associated with natural infection had been earlier described (Zamri-Saad et al. 2010). Therefore, there is need to properly understand the disease for its eventual control especially as regards the distribution of the bacterial antigen in tissues of the fish in acute and chronic infection with the use of PCR and IHC.

Infection through water is the most popular means of pathogen transmission in *Streptococcus* spp. infection and several transmission routes had been employed experimentally (Agnew and Barnes 2007) especially infection via IM and IP routes. However, there is very little information on the susceptibility to infection when there is skin abrasion, which is a possible scenario on the field (Chang and Plumb 1996; Shoemaker et al. 2000). This study therefore evaluates the possible role of skin cut in the transmission of *S. agalactiae* and it also described the clinicopathological changes and the detection of *S. agalactiae* antigen in the tissues of experimentally infected red tilapia by routine bacterial isolation, PCR and IHC.

## Materials and methods

### Fish

A total of 192 red tilapia (*Oreochromis* sp*.*), apparently healthy fish were obtained from an Aquaculture Extension Centre (AEC), Department of Fisheries Malaysia, Bukit Tinggi, Pahang. Fish with weight between 100-150 g were selected for the study. Prior to experiment, tanks were disinfected and cleaned. The source of water was dechlorinated and continuously aerated. Water quality was monitored and maintained throughout the experiment. The fish used, were screened for bacteria (particularly for *S. agalactiae* and external parasites). The fish were fed *ad libitum* with commercial feed before the experiment but throughout the experiment, the fish were off feed.

### Bacteria

*S. agalactiae* isolated from previous outbreak was used for the study (Siti-Zahrah et al. 2005). The organism was maintained in stock agar and also in glycerol stock. The bacteria stocked in nutrient agar were subcultured onto blood agar (Oxoid, UK) and incubated at 30°C for 24 h (Evans et al. 2004). The brain heart infusion broth (BHIB, Oxoid, UK) was used to subculture the colony and incubated in shaker incubator at 30°C for 24 h. A serial dilution and standard plate count techniques were used to determine the bacteria concentration (Alcamo 1997). 0.1 ml from the highest dilution was poured and spread onto the blood agar and incubated at 30°C for 24 h. Between 25 to 250 colonies were counted before the concentration was expressed as colony forming unit per millilitre (CFU/ml). The last concentration of live *S. agalactiae* used for inoculation was 10^9^ CFU/ml.

### Experimental design

The experiment was done by challenging the fish with 10^9^ CFU/ml of live *S. agalactiae* using different routes of infection. The fish (n=192) were divided into four groups with duplicates. Group 1 (n=24) were exposed to *S. agalactiae* through intraperitoneal (IP) route and transferred into the 6 L tank, Group 2 (n=24) were exposed to *S. agalactiae* through immersion bath (IM) (5 L of water + 1 L of *S. agalactiae* broth) for 10 min before being transferred into the 6 L tank and Group 3 (n=24) were exposed to *S. agalactiae* through immersion bath (5 L of water + 1 L of *S. agalactiae* broth) for 10 min with the body of fish being incised (IC) (0.5 cm) at the caudal part before being transferred into the 6 L tank. Group 4 (n=24) was the control unchallenged group. The fish were kept for 24 h off fed and the clinical signs were observed continuously within 24 h duration of the experiment. The gills, brain, eyes and kidney were collected from three fish four hourly within 24 h. The tissue samples were subjected to bacterial culture, PCR, histopathology and IHC.

### Bacteria isolation and identification

#### Bacteria culture

The swab from the organs that were collected four hourly were immediately streaked onto the blood agar plates (Evans et al. 2004) and incubated at 30°C for 24 h. Gram staining were performed to identify Gram-positive cocci in chain or paired and catalase test negative organisms. Finally, the colonies were further characterized using the commercialized test kit, API rapid ID 32 Strep® (bioMerieux SA, Marcy I’Etoile, France).

#### PCR

For confirmation of *S. agalactiae*, total cellular DNA was extracted using Wizard Genomic DNA Purification Kit (Promega, USA) according to manufacturer’s protocol. The extracted DNA was then further evaluated by PCR for *S. agalactiae*-specific section of 16S-23S rRNA intergenic spacer region with primers STAUR 4 [ACG GAG TTA CAA AGG ACG AC] and 6 [AGC TCA GCC TTA ACG AGT AC], and cycling conditions described as follows; 1 cycle at 94°C for 4 min, followed by 34 cycles at 94°C for 1 min, 52°C for 1 min, 72°C for 1 min and finally elongation at 72°C for 10 min. Seven μl of the amplified products was electrophoresed using 1.0% (w/v) agarose gel in 1× TBE electrophoresis buffer (0.1 mM Tris/HCI, 0.1 mM boric acid, 0.002 mM EDTA, pH 8.3) (Sambrook et al. 1989). The gel was stained with GelRed Nucleic Acid Gel Stain (Biotium, USA).

#### Histopathology

Fish were sacrificed with pitting technique, where the gills, eye, brain, and kidney tissue samples were collected every 4 hours post challenge (hpc), then fixed in 10% formaldehyde, and processed using a standard histological technique. After 24 h fixation, the tissue was trimmed before dehydrated in an ethanol series, followed by embedding in paraffin, and finally serial sectioning at 4 μm. The sections were stained routinely with haematoxylin and eosin (HE). Histopathological changes in each organ were semi quantitatively scored as none (0), mild (1), moderate (2), or severe (3).

#### IHC

Serial 4 μm thick sections were cut from the paraffin-embedded tissue blocks onto silane-coated glass slides. The slides were dried for 15 min at 56-60°C, dewaxed in xylene and rehydrated through a graded alcohol series. The slides were washed with PBST for 10 min. Endogenous peroxidase activity was blocked with freshly prepared 3% hydrogen peroxide for 5 min in room temperature and rinsed and washed with PBST for 2 min. In enhancing the tissue to be immunoreactive, heat-mediated antigen retrieval with citrate buffer solution in microwave oven was used. Sections were blocked with blocking buffer 1% normal serum (Bovine serum albumin) and PBST, then sections were incubated with tilapia anti *S. agalactiae* with the dilution of 1:50 for at least 1 h at 37°C in an incubator. Then the procedure was followed by rinsing and washing with PBST for 5 min. Sections were incubated again at 37°C for 30 min with secondary antibody (goat anti-tilapia) with the dilution of 1:100. The slides were rinsed and washed with PBST for 5 min before DAB was applied 1 ml diluents to a 1 drop DAB for colour change. Once the sections became brown, the slides were immediately rinsed with distilled water and the slides were stained using Mayer’s haematoxylin solution for the background colour.

### Statistical analysis

Statistical analyses were performed using MedCalc for Windows, version 12.2.1.0 (MedCalc Software, Mariakerke, Belgium) and tested at 5% level of significance. The differences in the data of lesion scoring were analyzed using Kruskal-Wallis and post-hoc tests were performed using Conover pairwise comparison test.

## Results

### Clinical and macroscopic findings

The clinical signs observed during 24 hpc with *S. agalactiae* demonstrated haemorrhage around the eyes, operculum, fin and/or body, erratic swimming, c-shaped body curvature, imbalance, some were dull and were isolated from the others. The clinical signs were most obvious in fish that were challenged IP followed by IC and lastly by IM bath of live *S. agalactiae*. The signs were first noticed as early as 2 hpc in the IP group, 4 hpc in IC followed by IM 8 hpc. At 4 hpc, the lesions observed include enlarged and congested spleen, pale and haemorrhagic liver, engorged gall bladder, congested kidney and softening of the brain (Table 1).

**Table 1:** **Semi quantitative scoring of eye, brain, and kidney in red tilapia,*O. niloticus*hybrid, challenge experimentally to different routes of infection**

Lesion		Brain	Eye	Kidney
		Infiltration of mononuclear cells, congestion, detachment of blood vessel, hydrophic degeneration	Haemorrhage, congestion, infiltration of mononuclear cells, widening of retina layer	Hydropic degeneration, congestion, haemorrhage,
Hour	Group			
4	1	3	2	3
	2	1	2	2
	3	1	1	2
8	1	3	2	3
	2	1	3	2
	3	1	2	3
16	1	3	2	3
	2	2	3	3
	3	2	3	3
24	1	3	3	3
	2	3	3	3
	3	3	3	3

### Mortality

There was no mortality due to the infection at the end of the experiment (24 hpc).

### Bacteria isolation and identification

The primary isolates from the sampled organs (eye, brain and kidney) revealed small pinpoint or minute transparent colonies on blood agar. The colonies were minute (0.5-1.0 mm), transparent, round and convex entirely. The colonies were characteristically presumptive of *Streptococcal* spp., Gram-positive cocci in chains, single or pairs and catalase negative. The biochemical profile of the isolates was confirmed by API rapid ID 32 Strep® (bioMerieux SA, Marcy I’Etoile, France) with 99.9% sensitivity to *S. agalactiae*.

Table 2 shows the bacterial isolation from tilapia samples for every 4 h sampling. *S. agalactiae* was isolated from twelve tilapia from IP group, ten fish from IC group and seven fish from IM group.

**Table 2:** **Results of microbiological examination of different groups and organs of red tilapia for*S. agalactiae***

	IP Group			IM group			IC group		
Hour	Brain	Eye	Kidney	Brain	Eye	Kidney	Brain	Eye	Kidney
C	-	-	-	-	-	-	-	-	-
4	+	+	+	-	+	-	-	+	-
8	+	+	+	-	+	-	+	+	-
12	+	+	+	-	+	+	-	+	+
16	+	+	+	-	+	-	-	+	-
20	+	+	+	-	-	-	-	-	-
24	+	+	+	-	+	-	-	+	-

Note: (−) negative isolation; (+) positive isolation; (C) control.

### PCR

**Figure 1:**
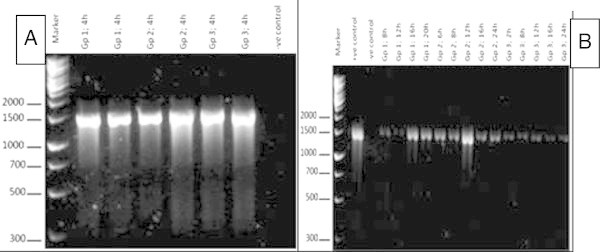
**A and B: Ethidium bromide-stained 1.5% agarose gel of multiplex PCR products.** All the samples *S. agalactiae* substrains yielded a single 1457-bp product, indicating the presence of the 16s ribosomal ARN gene sequence. Molecular size markers are shown on the left. (Gp, group; h, hour).

Based on Figure 1A and 1B, all the colonies that were subcultured were positive for *S. agalactiae* at 1457-bp*. S. agalactiae* were isolated in the target organs from the different routes of infection at every 4 till 24 hpc.

### Histopathology and lesion scoring

*S. agalactiae* induced acute inflammation in the brain, eye and kidney. Histological lesions were more severe in IP followed by IC and lastly IM throughout the 24 hpc. The meninges of telecephalon and cerebellum were infiltrated with inflammatory cells which in some cases were accompanied by haemorrhage and mild infiltration of eosinophilic granular cells which were located dorsal to the fourth ventricle. The lesions were more severe in IP12 (intraperitoneal 12 hpc) compared to IM12 (immersion 12 hpc) and IC12 (immersion cut 12 hpc). Kidney tissue showed various degrees of degeneration, tubular necrosis, haemorrhage and infiltration of the inflammatory cells especially lymphocytes. The lesions were more severe in IP12 than IM12 and IC12. The eye lesions were characterized by a mild cellular infiltrate, comprising macrophages and eosinophils which were observed in the choroid and periorbital tissues 12 hpc. The meninges were congested and thickened with the presence of eosinophilic granular cells in the brain. Based on the lesion score, there was no significant difference (*P* > 0.05) between the lesion induced by the routes of infection in most of the organs except the brain.

### IHC

**Figure 2:**
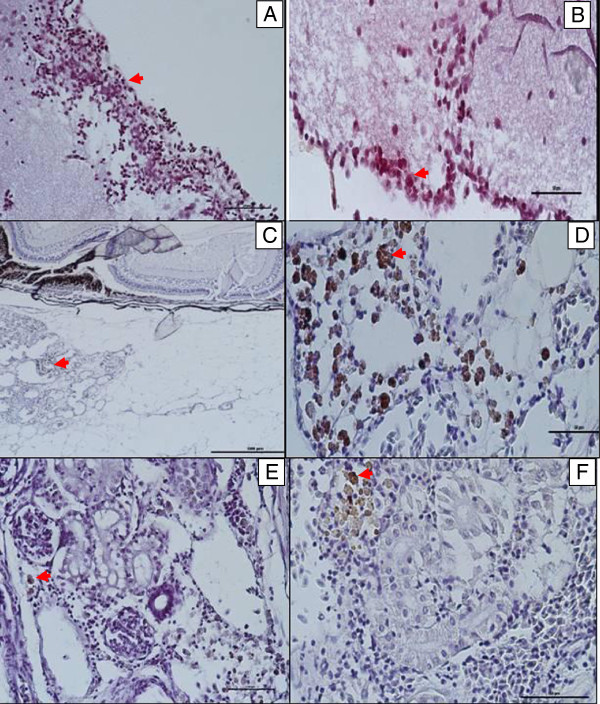
***Streptococcus agalactiae*antigens were found in A, B: the meninges and the meningeal blood vessel lumen and wall. C** and **D**: blood vessels and capillaries in the periorbital area containing macrophages. **E** and **F**: in the kidney; in the blood vessels lumens, walls and interstitial capillaries.

In IP and IC administration, the intense staining of *S. agalactiae* antigen was observed in the blood vessels lumen and walls. While in the kidney, it was observed in the interstitial capillaries, areas of haemorrhage and necrosis with cellular infiltration, some were observed in the macrophages. *S. agalactiae* antigens were detected most in the meninges, the meningeal blood vessel lumen and wall. In the eye, the choroid was more affected with the periorbital area containing macrophages with *S. agalactiae* antigens. The pattern of immunostaining of the tissues with *S. agalactiae* followed similar pattern in the IM group with less intensity. The antigens were observed in the tissues (brain, eye and kidney) harvested at 24 hpc in all the infected groups (Table 3; Figure 2).

**Table 3:** **Results of IHC examination of different groups and organs of red tilapia for*S. agalactiae***

Group	Brain	Eye	Kidney
C	-	-	-
IM4	-	-	-
IM12	-	-	-
IM16	+	+	+
IC8	-	-	+
IP4	+	-	+
IP8	+	+	+
IP12	+	+	+
IP16	+	+	+
IP20	+	+	+
IP 24	+	+	+

Note: (−) negative brown staining; (+) positive brown staining; (C) control.

## Discussion

This investigation describes the clinicopathological changes and the immunohistochemical detection of antigens in acute experimental *S. agalactiae* infection in red tilapia. The red tilapia challenged with *S. agalac*tiae showed various clinical signs and lesions that are consistent with the disease such as skin, fin and visceral organ haemorrhage/congestion while histopathological changes observed in the brain, kidney and eye are highly suggestive of septicaemia as observed in natural and experimental *Streptococcal* infection (Evans et al. 2002; Salvador et al. 2005; Suanyuk et al. 2005; Austin and Austin, 2007; Musa et al. 2009) with detection of bacterial in the tissues after 8 hpc.

The detection of *S. agalactiae* in tissues soon after infection further suggested the three organs as the target organ of the organism in the order of the brain, eye and kidney (Abuseliana et al. 2011). This observation was also reported for warm water streptococcosis associated with *S. iniae* in rainbow trout (Lahav et al. 2004). The gross pathological changes seen in this study were typical of septicaemia associated with *S. agalactiae* infection. The meningitis and blood vessels congestion were associated with loss of orientation and abnormalities often associated with *S. agalactiae* neurotropism (Eldar et al. 1995; Abuseliana et al. 2011).

Although most experiments showed that tilapia are more susceptible to *S. agalactiae* by IP route than IM, this study showed that IC which mimic the situation on field is not totally different from the IP group which further showed that minor cut of the skin possibly due to cannibalism or aggression or mild injury due to netting could lead to increased susceptibility to *S. agalactiae* infection in tilapia.

The bacteria were isolated at 4 hpc mostly from IP route in the brain, eye and kidney, followed by IC in the brain and eye lastly by IM in the brain only. This showed that brain is the primary target organ. The severity and wider distribution observed with IP route may be due to IP being a direct route of infection into the body when compared to the IC which had a means of entry into the body while IM has the natural skin defensive mechanisms to contend with. The effect on the blood vessel and subsequent detection of antigens in blood vessels, capillaries in the kidney and meningeal capillaries by IHC further lend credence to the report of Zamri-Saad et al. (2010) that vasculitis and thrombosis are some of the features of streptococcosis in tilapia.

There was no mortality within 24 h despite severe pathological changes observed within this period and this agreed with the report of Abuseliana et al. (2011) who reported mortality 48 hpc, while 50% of the challenge fish died on day 5 postchallenge. The fact that IP route had more lesion than IC and IM showed that the pathogenicity of the bacteria depends on level of septicaemia, with the brain being the primary target organ for *S. agalactiae* irrespective of the routes employed (Paperna 1996).

## Conclusions

*S. agalactiae* has been recognized as pathogenic to fish especially tilapia causing septicaemia and severe pathological changes in all different routes of infection (24 hpc) used in this study. The lesions are observed in the brain, eye and kidney and are more severe in the IP route, followed by IC and lastly IM. The detection of the antigen by IHC in the blood vessel lumen and on the wall which is described for the first time proves vasculitis and septicaemia as the major pathogenesis and it also explain the role of IC route in streptococcosis in red tilapia.
